# Reading comprehension intervention in populations with developmental language disorder: A scoping review

**DOI:** 10.1016/j.jcomdis.2025.106608

**Published:** 2025-12-04

**Authors:** Joseph Hin Yan Lam, Jiali Wang, Amy S. Pratt, Elizabeth D. Peña

**Affiliations:** a School of Education, University of California, Irvine, USA; b Department of Teaching, Learning, & Culture, Texas A&M University; c Department of Communication Sciences and Disorders, University of Cincinnati, Cincinnati, OH, USA

**Keywords:** Reading comprehension, Intervention, Developmental language disorder, Scoping review

## Abstract

**Purpose:**

Approximately 25–50 % of children with developmental language disorder (DLD) demonstrate difficulties in reading comprehension, which are associated with difficulty in language learning. Given the high likelihood that children with DLD will experience difficulties in reading comprehension, it is important to examine the effectiveness of interventions that target these challenges. The purpose of this study is to conduct a scoping review of the literature on reading comprehension interventions in populations with DLD to synthesize the existing literature and highlight knowledge gaps in supporting reading comprehension in this population.

**Methods:**

The five-step framework for scoping reviews described by Arksey and O’Malley (2005) was adopted to identify and evaluate literature focused on reading comprehension interventions for populations with DLD. Twenty-four studies met the inclusion criteria, with five randomized controlled trials, 12 group comparison studies, and seven single case studies or reports.

**Results:**

Identified interventions were aligned with the Active View of Reading (Duke & Cartwright, 2021), targeting active self-regulation, word recognition, bridging processes, and extended discourse. The majority of the included studies reported improvements in reading comprehension. All studies targeting vocabulary knowledge and morphological awareness found significant positive gains. However, several validity concerns emerged in the included studies, such as high variability in the intervention dosage and intensity and a lack of diversity in measurement methods. These issues could hinder the translation of the findings into clinical practice.

**Conclusion:**

The current review reveals several gaps: (1) underrepresentation of multilingual children with DLD and children speaking languages other than English as a first language; (2) insufficient examination of children with DLD’s performance across various genres and question types; and (3) a lack of understanding of optimal intervention dosage for reading comprehension intervention in this population.

## 1. Introduction

Developmental language disorder (DLD), formerly characterized as specific language impairment, language learning disorder, or primary language impairment, is a high-incidence neurodevelopmental disorder associated with difficulties in producing and understanding language (Bishop et al., 2017). DLD affects 7–10 % of children worldwide (Norbury et al., 2016; Tomblin et al., 1997), and approximately 25–50 % of children with DLD experience co-occurring reading difficulties, particularly in reading comprehension (Adlof & Hogan, 2018). These challenges are likely attributable to impairments in semantics, morphosyntax, and pragmatics (Castle et al., 2018). Children’s reading comprehension abilities can affect their engagement in learning, academic performance, and future career development (e.g., Dockrell et al., 2011; Nag, 2014). The current scoping review examines features of reading comprehension interventions in children with DLD, including an evaluation of intervention outcomes.

Reading comprehension is an extension of language comprehension that represents an integral component of speech-language pathologists’ (SLPs’) expertise. Literacy, including both reading and writing, falls within the scope of practice for SLPs worldwide (American Speech-Language-Hearing Association [ASHA], 2016; Health & Care Professions Council, 2013; Speech Pathology Australia, 2020; The Hong Kong Institute of Speech Therapists, 2022). The role of SLPs in the domain of literacy involves prevention of written language difficulties, reading and writing assessment, and delivery of interventions targeting difficulties with written language (ASHA, 2001). SLPs often report low confidence and self-efficacy in providing literacy-related services (Yi & Erickson, 2024a, 2024c), which may be attributable to limited training in literacy during graduate education (Yi & Erickson, 2024b). This scoping review fills an important gap in knowledge by summarizing the available evidence on reading comprehension interventions for populations with DLD and identifying potential areas of future research. Therefore, the purpose of the current scoping review is twofold: (1) to summarize the research about reading comprehension interventions in populations with DLD using the Active View of Reading as a framework for organizing the existing literature (AVR; Duke & Cartwright, 2021) and (2) to identify limitations and gaps in the available evidence base.

### 1.1. Theories of reading comprehension

Reading comprehension is a complex and multidimensional construct. There has been a preference towards parsimonious models of reading such as The Simple View of Reading (SVR; Gough & Tunmer, 1986; Hoover & Gough, 1990) which describes variability in reading comprehension across two component skills: decoding (or the act of mapping sounds onto symbols) and language comprehension (or the act of attaching meaning to the decoded words). According to this model, children with DLD, who by definition struggle with oral language, are also likely to struggle in reading comprehension, given their difficulties with language comprehension. However, the high levels of covariability between decoding and language comprehension suggest that other skills may exert influence on the reading process, above and beyond the two components of the SVR (Cartwright et al., 2020; Cutting & Scarborough, 2006; Foorman et al., 2020). Subsequent models of reading comprehension have attempted to account for these additional factors, including internal processes such as self-regulation (e.g., Kim, 2017) and external knowledge, such as familiarity with the text structure and/or genre (e.g., Hogan et al., 2011; Kim, 2017).

**Fig. 1.**
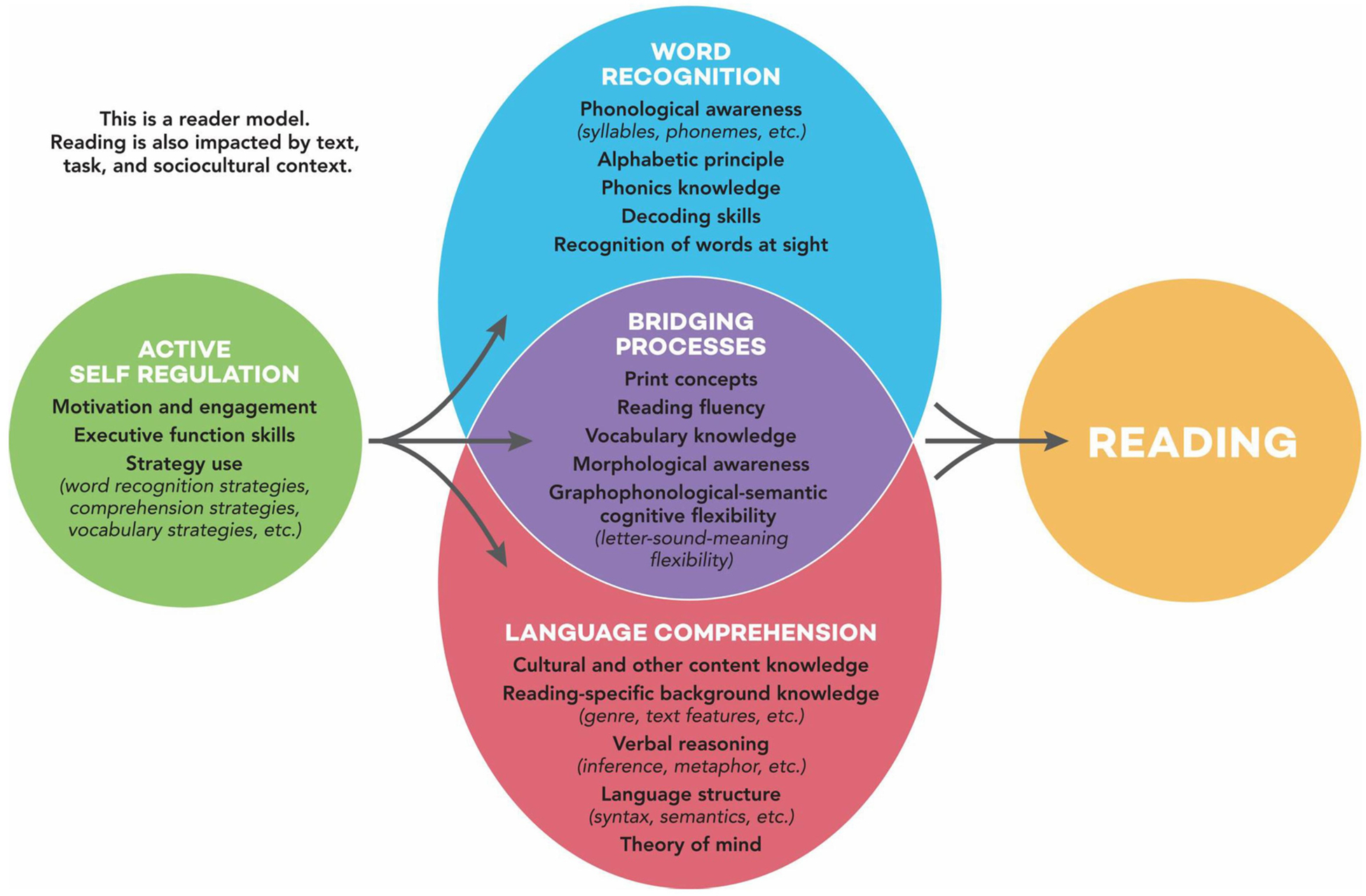
The Active View of Reading Model (Duke & Cartwright, 2021). *Note*. The reproduction of this figure is covered by a Creative Commons License.

The Active View of Reading (AVR; Duke & Cartwright, 2021), as shown in Fig. 1, is an expanded model of reading comprehension that builds upon the SVR. The AVR includes four components that explore the different component skills that affect reading comprehension: active self-regulation, word recognition, bridging processes, and language comprehension. In contrast to other models of reading comprehension, the AVR emphasizes that all of its components are instructionally malleable, thus making it ideal for guiding the development and implementation of reading comprehension interventions.

Given that the AVR represents a relatively recent expansion of the SVR, research exploring its application to diverse populations is limited. With respect to readers of diverse orthographies, we could not identify any empirical studies validating its use across orthographic types. However, there is a substantial body of research showing that the SVR is valid and applicable for beginning readers of transparent (non-English) alphabetic orthographies (see Florit & Cain, 2011, for a meta-analyses; and more recently, Silinskas et al., 2024, for a discussion of the SVR in Lithuanian) as well as opaque logographic orthographies (e.g., deep version of Arabic orthography, Asadi & Ibrahim, 2018; Chinese, Joshi, 2018). At both ends of the orthographic spectrum, significant variance in reading comprehension was explained by decoding and language comprehension, though the relative influence of the two components was different for readers of different orthographic types.

There is emerging evidence validating the AVR for children with neurodevelopmental disorders, such as DLD. A previous meta-analysis showed that interventions targeting the four components of the AVR yielded medium to large effect sizes on reading comprehension in children with reading difficulties (Burns et al., 2023). However, this study did not disentangle the source of children’s difficulties in reading comprehension. Children with DLD represent a distinct subgroup of struggling readers with a unique profile of strengths and challenges, which may, in turn, require a different set of intervention targets. Most notably, children with DLD are at higher risk for reading comprehension difficulties even when decoding skills are adequate (Bishop et al., 2017; Catts et al., 2005). Compared to children with dyslexia or struggling readers, children with DLD often show broader impairments in vocabulary, morphosyntax, and discourse skills that underpin text comprehension (Nation et al., 2004; Snowling et al., 2016). A more recent systematic review demonstrated the applicability of the AVR in explaining reading comprehension ability in children with DLD (Lam et al., 2024). Consistent with the AVR, results of the review showed strong predictive relationships between language-based measures (vocabulary, listening comprehension, oral language comprehension, grammatical knowledge) and reading comprehension among children with DLD.

### 1.2. Sources of reading comprehension difficulties in children with DLD

The AVR can potentially guide reading comprehension interventions for children with DLD. The model suggests that active self-regulation is the basis for reading comprehension, as it influences all other components of reading comprehension. Active self-regulation refers to the cognitive skills that help readers focus on what they are reading, including executive function skills (attention, inhibition), meta-cognitive strategy use, and motivation and engagement (Duke & Cartwright, 2021). When applying these skills, children deliberately utilize strategies to construct meaning from the text, and they maintain motivation and engagement to stay on task. Reading comprehension interventions targeting self-regulation skills are important for reading comprehension due to the dynamic nature of how reading comprehension is achieved. Reading comprehension is a goal-oriented process where readers use executive function skills to make inferences, monitor comprehension, and engage in the reading process. A previous meta-analysis showed that interventions targeting active self-regulation can improve reading comprehension in average readers, as compared to interventions focusing solely on word recognition and language comprehension (Burns et al., 2023). Because it is well-documented that children with DLD have lower performance across various aspects of executive function skills, including interference control and working memory (e.g., Blom & Boerma, 2020), it is plausible that they employ lower self-regulation during reading comprehension. Thus, reading comprehension interventions that target self-regulation and strategy use may have the potential to improve reading comprehension skills for children with DLD.

The AVR’s word recognition and language comprehension components are consistent with the Simple View of Reading. Word recognition was originally described in the SVR model as decoding, which includes both decoding via one’s knowledge of sound-symbol correspondence and the automatization of word recognition (Gough & Tunmer, 1986; Hoover & Tunmer, 2020). Children with DLD often have difficulty in phonological processing (Catts et al., 2005; Snowling et al., 2019). These difficulties may impact serial recognition and the production of speech sounds, which can in turn hinder word recognition when reading. Thus, children with DLD may stand to benefit from phoneme- and word-level intervention to help improve their reading comprehension abilities.

Language comprehension refers to a set of skills ranging from language skills, such as syntactic skills to broader cultural and world knowledge (Duke & Cartwright, 2021). Language comprehension is a major area of weakness among children with DLD (Bishop et al., 2017). These may include difficulties in verbal reasoning, syntax, and theory of mind (Montgomery et al., 2018; Nilsson & de Lopez, 2016). Interventions targeting language comprehension in children with DLD, as shown in previous meta-analyses, are operationalized as discourse-level interventions using narrative, listening comprehension, and conversational skills (Cleave et al., 2015; Winters et al., 2022). These extended discourse interventions aim to enhance comprehension of sentences, inference skills, and knowledge of text structure. Interventions targeting language comprehension might also have a generalization effect on reading comprehension outcomes in children with DLD.

The bridging processes component is the final component of the AVR model. It addresses the overlapping skills between word recognition and language comprehension that were not accounted for in the SVR framework (Duke & Cartwright, 2021). Specifically, vocabulary knowledge and morphological awareness are considered bridging processes in the AVR, because both word recognition and language comprehension skills are required. Children with DLD have significant impairments in semantics and morphosyntax (Kan & Windsor, 2010; Krok & Leonard, 2015). As such, interventions targeting vocabulary knowledge in children with DLD are relatively well-studied and have been conducted across a wide range of ages, spanning preschool to adolescence (Cirrin & Gillam, 2008; Lowe et al., 2018). Vocabulary interventions in children with DLD have employed a variety of strategies, including enhancing phonological awareness to improve the letter-sound correspondence (e.g., Gillam et al., 2008), leveraging morphological awareness to improve word definitions and word classes (e.g., Katz & Carlisle, 2009), and embedding vocabulary instruction within highly contextualized books and conversational speech (e.g., Joffe et al., 2019). The diversity of treatment approaches echoes the overlapping and interconnected nature of the bridging processes component. By strengthening semantic networks, these interventions enhance children’s ability to understand the meaning of the text during reading. Considered collectively, the prior literature on DLD suggests that children with language-learning disorders may experience difficulties across all four components of the AVR, which may impact their reading comprehension. However, to date, no study has systematically summarized the features of interventions targeting reading comprehension in this population. Thus, the current study aims to examine intervention studies conducted with children with DLD to evaluate the latest evidence for improving reading comprehension and to identify current gaps in the literature.

### 1.3. Considerations for examining reading comprehension intervention research

The variability among interventions documented in the existing literature presents significant challenges for health practitioners, educators, and policy makers in interpreting the evidence. To address these challenges, an international consortium of experts in speech and language pathology identified key barriers to evaluating intervention research within the field, focusing specifically on dosage, context, and outcomes (Frizelle et al., 2023).

A major challenge in evaluating the evidence for intervention research is dose form and dosage-related information. Dose form is defined as the experimental manipulation of the intervention technique, helping researchers to determine the optimal dosage and establish causal inference between the intervention and the outcome (Frizelle et al., 2023). Given that reading comprehension involves multiple subcomponents (Catts & Kamhi, 2017), the dose form and technique adopted in the intervention may consist of multiple skills addressing multiple aspects of reading comprehension. In this case, it is important for clinicians to identify the specific skills that the intervention is targeting. Furthermore, a quantitative description of dosage is crucial for implementation, given differing strengths of associations between reading comprehension and its component skills. This includes dose (i.e., the number of teaching episodes delivered within a session), dose frequency (i.e., number of doses divided by unit time), and total intervention duration (Warren et al., 2007).

Outcome measures can also impact effect sizes and the statistical significance of interventions. Reading comprehension is commonly measured using both standardized and non-standardized measures. Standardized reading measures are designed to describe a child’s overall reading ability and provide psychometric information, such as internal reliability, test-retest reliability, and validity, as described in test manuals. In contrast, non-standardized measurements are usually designed to measure targeted reading behaviors and are often more proximal to the intervention targets. A prior meta-analysis on reading comprehension interventions identified measurement type (i.e., standardized versus non-standardized tests) as a significant moderator of intervention outcomes, with non-standardized tests demonstrating larger intervention effect sizes than standardized tests (Filderman et al., 2022). In addition, standardized tests select features that differentiate between typical and atypical processing, which might not be comprehensive enough to capture all aspects for tracking intervention outcomes (McCauley & Swisher, 1984). Thus, it is important to review the content and scope of the assessment measuring reading outcomes to examine whether it can effectively capture change over time.

Intervention context is another critical aspect to consider. Home-based and school-based interventions differ in terms of types of available support, student motivation, and implementation challenges. According to self-determination theory (Krapp, 2005), the school environment has higher collaboration between professionals and greater peer support compared to the home environment. School is also a common intervention context for literacy support. On the other hand, intervention in the home context enables parental support throughout the intervention, and family has a strong impact on early child literacy development through parental involvement in reading and enhancing children’s reading motivation (Loera et al., 2011; McElvany & Artelt, 2009).

### 1.4. Research aims

Although there is a recent meta-analysis on reading comprehension interventions using the AVR (Burns et al., 2023), this has yet to be extended to include children with DLD. Because children with DLD have specific difficulties in the subcomponents of reading, such as language skills (language comprehension, bridging processes), word recognition, and active self-regulation, the target skills and the dosage of intervention for this population may differ from the general population. It is thus meaningful to examine the needs of this specific population in reading comprehension. The aims of the current study are:

1. To synthesize the existing literature about reading comprehension interventions in populations with DLD using the AVR framework.
2. To identify potential limitations of existing reading comprehension interventions in populations with DLD.
3. To identify potential research gaps in reading comprehension interventions in populations with DLD.

## 2. Method

### 2.1. Scoping review framework

The current study adopted the scoping review framework proposed by Arksey and O’Malley (2005). The framework includes five stages of the review process, including (1) identification of the research question, (2) identification of relevant studies, (3) selection of relevant studies, (4) charting of findings, and (5) reporting of results. This framework was chosen given that it can illustrate the field’s interest in a rigorous and transparent manner, identify gaps in the literature and summarize the research findings (Arksey & O’Malley, 2005). Furthermore, this framework has been adopted in the field of speech-language pathology related to clinical areas with limited research (e.g., Tucci & Choi, 2023).

### 2.2. Identification of research question

The current review was motivated by the following research question: What is known from the existing literature about reading comprehension interventions in populations with DLD? In order to answer that question, we summarize the existing evidence base, as well as gaps and limitations in the existing literature. Though this question requires the synthesis of the evidence regarding efficacy and effectiveness, our primary purpose was to map the breadth, characteristics, and limitations in the literature on reading comprehension interventions for children with DLD. Our methodology aligns with the definition of a broad-scope review (Chandler et al., 2019) and seeks to include all available evidence—regardless of differences in designs, outcomes, and reporting style—in order to describe the existing literature and identify areas for future research, which might later support a systematic or meta-analytic review.

The present scoping review is pre-registered on the Open Science Framework Registries (Name: Reading comprehension intervention in children with DLD: A scoping review; Registration number: https://osf.io/mnc7t/).

### 2.3. Identification of relevant studies

Relevant studies were located in three electronic databases, including ERIC, EBSCO, and PsycINFO. The literature search on these databases was performed on Aug 7, 2022, and included all relevant studies through this date. The search strings combined terms for intervention, training, or treatment; language disorder, impairment, or disability; and reading comprehension. Complete search strings are available in Supplementary Material 1. Results of the literature search were then exported as RIS files and imported into Covidence systematic review software for screening and extraction (Covidence, 2023).

The following five criteria were applied for the eligibility criteria for inclusion in the current scoping review: (1) included a DLD or language-impaired group, which can be identified by SLPs at research, clinical or school settings, standardized assessments, or from any formal clinical records; (2) included a treatment or intervention component; (3) included at least one reading comprehension measure as an outcome or generalization measure in post-intervention or follow-up assessment; (4) published in a peer-reviewed journal and included original empirical findings; (5) published in English.

### 2.4. Study selection

The initial search generated 4867 references, which included 968 duplicates. 3894 unique references were imported to Covidence for the screening and extraction processes. The first author performed the title and abstract screening for all references, while the second author performed a second rater reliability check on 20 % of the references. The interrater reliability was 96.41 % (753/781) and inconsistency was resolved by the third author.

**Fig. 2.**
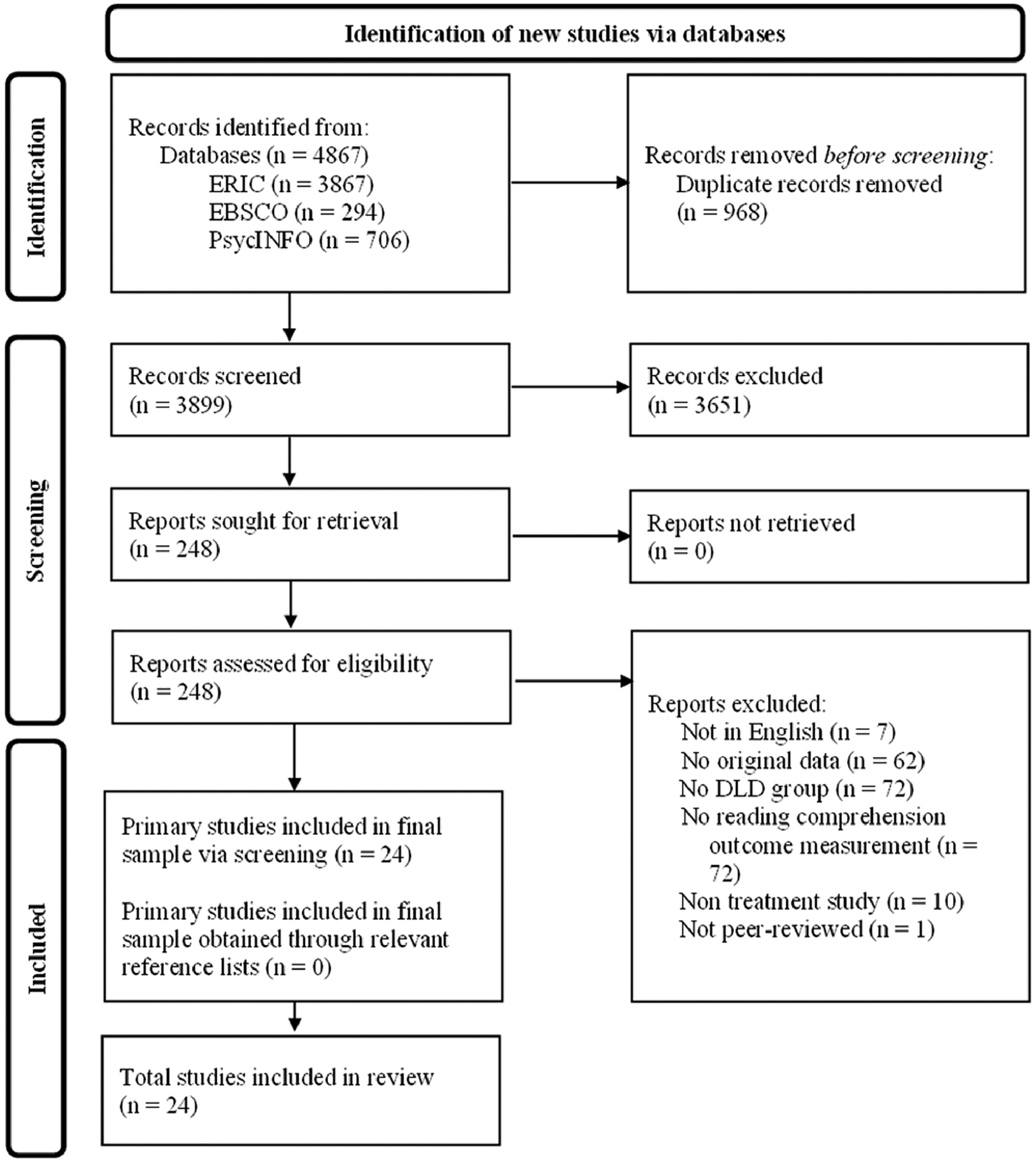
Search and Selection Process.

A subset of 248 articles was further screened at the full-text level using the criteria mentioned above. The first author screened all full text articles, while the second author performed a second rater reliability check on 20 % of them. Interrater reliability was 96 % (48/50). Inconsistencies were resolved by the third author. After reviewing all the articles, 24 articles were identified that met the inclusion criteria. Reference lists of the included articles were screened, and no additional articles were identified. Thus, 24 studies were ultimately included in the current systematic review. Fig. 2 shows the search and selection process.

### 2.5. Charting of findings

Study information was extracted for synthesis by the first author on all 24 included articles into a Google Sheet. There were three major areas for data extraction: participant characteristics, intervention characteristics, and intervention outcomes. The participant characteristics extracted included the sample size, sex, age (both mean and range), race, monolingual/bilingual status, and language(s) spoken by the participants. In addition, the terminology and the identification process of the DLD group were also recorded.

Regarding the intervention characteristics, duration, interventionist, setting, dose frequency, dosage, intensity, service delivery, participant size, social validity, and fidelity of the intervention were coded. Moreover, the description of the intervention was extracted together with language skills targeted in the intervention. Coding of language skills was based on the AVR (Duke & Cartwright, 2021). Four domains were included in this model: active self-regulation, word recognition, bridging process, and language comprehension (specific skills were specified within the four domains as shown in Fig. 1). For interventions that had multiple language domains/skills targeted, the first and second authors read the paper together and decided the primary domains/skills and other targeted language domains/skills were also noted.

The extracted intervention outcomes consist of the reading outcome measures and the statistics reported. The reading measurements adopted in the studies were extracted with the information of the reading text(s), question type(s), answer method(s), and reliability. For the statistics reported, the mean and standard deviation of the reading measurements were extracted at pre-test, post-test, and follow-up assessment if they were reported. The statistical analysis adopted to measure the difference in reading comprehension ability was extracted with the statistical test(s) conducted, statistical coefficient(s), and the significance level. The second author randomly completed the extraction 25 % of the included articles (6/24) and interrater agreement was 92.07 % (279/303). Discrepancies were resolved before coders moved into independent coding. The complete dataset is available in the replication materials (https://osf.io/5vs7n).

### 2.6. Intervention biases

The source of bias in literature reviews has received increasing attention. Thus, it is important to consider the risk of bias in the included studies rather than only summarizing the characteristics in the synthesis (Page et al., 2021). To assess the risk of bias, Quality Assessment of Controlled Intervention Studies, Quality Assessment Tool for Before-After (Pre-Post) Studies With No Control Group, and Quality Assessment Tool for Case Series Studies were adopted to assess the risk of bias for included studies based on the types of intervention design (National Institutes of Health, 2015). The first author completed the coding for all included studies, while the second author performed a second rater reliability check on 25 % of the included articles (6/24). The exact agreement for the risk of bias assessment was 95.71 % (67/70).

## 3. Results

### 3.1. Study characteristics

Results included in the final synthesis represent research across seven countries between 1988 and 2022 with half of them conducted in the US (12/24; 48 %). All interventions reported were conducted in English, except one intervention was conducted in Finnish and one intervention was conducted in English with Spanish support. Over half of the studies (13/24, 54 %) were conducted in the last ten years (2013–2023). Studies adopted various terms in characterizing the DLD groups (details described in the section below). DLD was used in the current study for clarity of reporting. Participants with DLD included in these intervention studies ranged from 1 to 394 and the age range was from 3.83 to 14.25 years old.

### 3.2. Participants characteristics

Regarding the age of the participants in the intervention, three of the included studies (13 %) targeted children below age of five (i.e., pre-reading stage). The majority of the included studies included participants from age five to ten (17/24; 71 %), which was the *learning to read stage*. Four studies (17 %) included adolescents as participants (i.e., *reading to learn* stage). Regarding the language(s) spoken by the participants, the majority of the studies reported participants were monolingual English speakers or first-language English speakers (21/24; 88 %). Two studies (8 %) included bilingual Spanish-English participants and one study (4 %) included first-language Finnish participants.

Regarding the description of DLD, 14 included studies (58 %) adopted the terms of language impairment or language disorder/deficits/difficulties (LI), followed by specific language impairment (SLI; *n* = 2; 8 %), weak language skills (*n* = 5; 21 %), and language-learning disabilities (LLD; *n* = 2; 8 %). Regarding the identification of the DLD group, 12 of the included studies (50 %) identified participants as DLD with speech-language pathologists’ diagnosis, followed by use of standardized tests (*n* = 9; 38 %), and use of screening tools (*n* = 4; 17 %). Note that for some studies, the participants were characterized as having LI or weak language skills, in which some of them were qualified as DLD (Sanabria et al., 2022; Wright et al., 2015). For Staudt (2009), results were only reported from participant Will, as he was the sole participated diagnosed with a language disorder.

### 3.3. Intervention characteristics

#### 3.3.1. Intervention context

The majority of the intervention studies (18/24; 75 %) were conducted at schools, followed by clinics (1/24; 4 %) and participants’ homes (1/24; 4 %). Four studies did not report the setting of the intervention programs. Eight studies (33 %) were conducted by researchers, followed by trained students (5/24; 21 %), teachers (4/24; 17 %), teaching assistants (4/24; 17 %), trained students (4/24; 17 %), computer programs (2/24; 8 %), and SLPs (1/24; 4 %).

#### 3.3.2. Intervention design

Five of the included studies (21 %) adopted a randomized control trial (RCT) design. Twelve included studies (50 %) used group comparison design, and the remaining seven included studies (29 %) adopted a single-case design. For the studies that adopted RCT and group comparison designs (*n* = 17), eight studies compared a reading intervention group with a waiting control, consultation, or no treatment group, while the other nine studies compared two or three active reading comprehension intervention groups. Thus, there were a total of 37 active reading comprehension intervention programs conducted with participants with DLD. A majority of the interventions (31/37; 84 %) were face-to-face as the service delivery method, whereas the remaining six interventions (16 %) adopted electronic devices to conduct the intervention. Twelve of the interventions (32 %) adopted individual therapy, 11 adopted group therapy (30 %), and nine of them adopted a mix of individual and group therapy (28 %).

#### 3.3.3. Intervention length

Regarding the active reading comprehension intervention programs reported (*n* = 37), the duration of the interventions ranged from three weeks to a school year (*m* = 12.24 weeks; *SD* = 10.52 weeks) and the dosage (total hours) of the interventions ranged from 2.5 h to 83.33 h (*m* = 22.31 h; *SD* = 23.11 h). Two to four times of interventions a week and daily intervention were the most commonly adopted dose frequencies (14/37; 38 %), followed by weekly intervention (9/37; 24 %). The intensity of the intervention ranged from 40 to 250 min per week (*m* = 75.95 min; *SD* = 62.76 min). See Table 4 for details about intervention length.

#### 3.3.4. Intervention target based on the AVR

The skills targeted in the intervention programs reported can be categorized into four major skills, namly self-regulation (*n* = 11), decoding (*n* = 13), vocabulary knowledge (*n* = 4), and extended discourse (*n* = 9), with reference to the AVR framework. Only vocabulary knowledge was a primary targeted skill among all skills in the bridging process under the AVR, so vocabulary knowledge was used as a category. Extended discourse was used as a category, replacing language comprehension in the AVR, to specify that both receptive and expressive language were targeted in the interventions. A majority of the intervention programs (22/37; 59 %) targeted multiple skills, while 15 of them (41 %) targeted only one skill throughout the interventions.

##### 3.3.4.1. Self-regulation.

Table 1 summarizes the interventions targeting self-regulation. One group-designed study focused on executive function skills; six group-designed studies and two single-case studies focused on different strategy use. These interventions recruited older children with DLD. Ritter, Colson et al. (2013) compared the effects of the 48-hour language and reading intervention with a four-hour Interactive Metronome addition, which focused on temporal processing and could improve reading comprehension. Participants were 49 nine-year-old children with DLD, and results showed the Interactive Metronome addition had higher performance on reading rate, reading fluency, and reading comprehension.

There were six group-designed studies and two single-case studies focused on the use of different strategies, which described nine different intervention programs and one replication. Intervention programs taught multiple reading strategies to children with DLD. Wright et al. (2015) implemented a 4-week reading intervention targeting six reading strategies with reciprocal teaching to 10, 13-year-old adolescents with DLD, and results showed a large effect size improvement in reading comprehension. This intervention was then replicated in the same study with 18, 13-year-old adolescents with DLD, and the results showed a large effect size improvement in word reading and reading comprehension. Takala (2006) provided a 5-week classroom-level reading strategies intervention, which included four cognitive reading strategies with reciprocal teaching, to 50 fourth- and sixth-grade Finnish students with DLD, and results showed gains in reading comprehension but did not reach statistical significance. McCartney et al. (2015) implemented a universal reading comprehension intervention for six- to eight-year-old typically developing children and children with DLD, targeting multiple text comprehension strategies in the routine classroom reading curriculum. Results showed a significant improvement in reading comprehension in both groups. Lukin and Estraviz (2010) taught multiple text comprehension strategies during the Reading Recovery program to six children with DLD for 21 weeks and found children reading book levels reached age-matched levels after the intervention.

Imagery training was the focus of two studies with three different intervention programs. Sanabria et al. (2022) adopted the EMBRACE program (Enhanced Moved by Reading to Accelerate Comprehension in English) for 56 second-to-fifth grade Spanish-English bilingual children with DLD, which involved the creation of multimodal simulations, including picture manipulation and imaginary picture manipulation. While the overall intervention effect was not significant, the intervention had a significant effect on comprehension of narrative and easier texts. Joffe et al. (2007) implemented a three-week imagery training program to nine 10-year-old children with DLD, and significant improvement was reported in literal and inferential reading comprehension questions.

There was one study related to the use of think-aloud strategy. Green and Roth (2013) conducted a single case study on intervention focusing on teaching “puzzle question” strategy under think-aloud condition to a fourth-grade student with DLD and found improvement in a standardized reading comprehension test.

A randomized controlled trial focused on a classroom-level intervention on information processing strategies, modifying teachers’ language input and direct vocabulary instruction (Starling et al., 2012). A total of 21 Grade 8 adolescents with DLD participated in the intervention group. Results showed that despite significant differences in reading comprehension between pre-post tests, the intervention group had no significant difference compared to the control group.

Overall, these studies provide early efficacy data on interventions targeting reading comprehension that incorporate self-regulation studies. Generally, pre- to posttest on reading comprehension measures are reported. But there is a need for an RCT to compare the efficacy of different strategies and determine the optimal dosage.

##### 3.3.4.2. Word recognition.

Table 2 summarizes the interventions targeting word recognition. There were seven group-designed studies and one single-case study focused on decoding skills, phonemic awareness, phonological awareness, letter-sound knowledge, and word reading. The majority of these interventions included younger children. There were two intervention programs primarily focusing on decoding skills. Crowe (2005) compared a 10-hour intervention focusing on decoding and vocabulary with an intervention using communicative reading strategies with eight-to-eleven-year-old students with DLD. Findings indicated that the intervention using communicative reading strategies had significantly higher gains on standardized and non-standardized reading measures. In an earlier study, Crowe (2003) found that participants who received intervention using communicative reading strategies had significantly better reading comprehension performance than the participants who received intervention focusing on decoding and vocabulary and the control group.

There were two studies focused on comparing different intervention programs targeted at phonemic awareness. Loeb et al. (2009) compared three different intervention programs, including Fast ForWord computerized intervention, computer-assisted language intervention, and individualized language intervention, with 103 six-to-nine-year-old children with DLD. All the interventions focused on phonemic awareness and language structures with differences in time allocation. There were no significant changes in reading comprehension skills in the maintenance test. Pokorni et al. (2004) compared three different intervention programs focusing on phonemic awareness, including Fast ForWord intervention, Earobics Step 2, and Lindamood Phonemic Sequencing Program, on 54 nine-year-old children with DLD. The Fast ForWord intervention and Earobic Step 2 were computerized programs, whereas the Lindamood Phonemic Sequencing Program was a face-to-face group intervention. Findings indicated no significant difference in reading comprehension between pre- and post-tests.

There were two studies focused on phonological awareness. Ritter, Park et al. (2013) reported a 12-week instruction of phonological awareness and sound-symbol correspondence to 34 students in Grades 1 to 3 with DLD, and the participants had significantly higher gains in word decoding and read comprehension than the control group (*n* = 30). Bowyer-Crane et al. (2008) compared an intervention focusing on phonological awareness, letter-sound knowledge, and reading with an oral intervention focusing on expressive language, listening skills, vocabulary, and inferencing on 152 6-year-old children with DLD for 20 weeks. There were no significant differences in reading comprehension ability in post-test scores between the two groups.

There was one study that reported intervention programs focused on letter-sound knowledge. Kouri (2016) compared the use of graphophonemic feedback strategies, meaning-based strategies, and combination of phonemic and meaning feedback strategies on reading comprehension in 15 seven-to-nine-year-old children with DLD. It showed that there were no significant differences in reading comprehension between feedback strategy types.

Buck et al. (1988) implemented a home tutoring program focusing on word recognition, print knowledge, and expressive language for eight seven-to-ten-year-old children with DLD for 15 weeks. The results showed significant improvement in reading comprehension.

Overall, there were mixed results on the efficiency of the pre- to post-tests of reading comprehension interventions that incorporate word recognition skills. Decontextualized and computerized programs showed limited changes in reading comprehension ability compared to meaning-based integrated interventions. Further work is needed to investigate the efficacy of phonological awareness and word recognition interventions with larger sample sizes and more rigorous design.

##### 3.3.4.3. Vocabulary knowledge.

Table 3 summarizes the interventions targeting vocabulary knowledge. There were four single-case studies focused on building vocabulary knowledge as the primary skill. Two focused on keyword knowledge, whereas the other two focused on the morphology of vocabulary. Rousseau et al. (1993) provided a 10-week intervention focusing on discussion of the meaning of 10 key words and listening preview (pointing to the words while listening) of the narrative passages to five 12-year-old Spanish-English bilingual adolescents with DLD and found that the combination of both conditions yielded higher performance than the baseline period. Staudt (2009) reported an intervention on expanding vocabulary knowledge through building connections between vocabulary and understanding multiple meanings of vocabulary during poem reading to a fourth-grade child with DLD and found improvement in word reading, reading speed, and reading comprehension after a school year.

Two interventions on morphology targeted at building up understanding of vocabulary. Katz and Carlisle (2009) provided a combination of derivational morphological analysis and context analysis in a 12-week intervention to three fourth-grade students with DLD and found small to large effect size improvement in reading comprehension. A 6-year-old child with a history of DLD received a 7-week both derivational and inflectional morphological awareness intervention on 12 targeted affixes and showed gains in reading comprehension but did not reach clinically relevant levels (McLeod & Apel, 2015).

Overall, there were improvements and early efficiency on the pre- to post-tests of reading comprehension interventions focusing on vocabulary knowledge. There is a need for group design with a larger sample size to confirm the efficacy of these interventions.

##### 3.3.4.4. Extended discourse.

Table 4 summarizes the interventions targeting extended discourse. In addition to the five reading intervention programs mentioned in the self-regulation and decoding sections, which served as active comparison groups, there were three other studies reporting three different reading comprehension intervention programs focusing on extended discourse. These studies investigated extended discourse intervention on reading comprehension in young children. Fricke et al. (2013, 2017) reported a 30-week Nuffield Early Language Intervention program focusing on narrative skills, vocabulary, active listening, and expressive language to 4-year-old children with DLD. Fricke et al. (2013) reported a significant generalization effect of the intervention on reading comprehension in the maintenance test, whereas Fricke et al. (2017) reported no significant difference in reading comprehension in the maintenance test compared to the waiting control group at Grade 1. Fricke et al. (2017) also included a 20-week Nuffield Early Language Intervention program for 5-year-old children with DLD at kindergarten and found no significant difference in reading comprehension compared to the 30-week intervention group and the waiting control group at Grade 1. Revees et al. (2019) implemented Talk Boost KS2, which focuses on active listening, vocabulary, narrative skills, conversational skills, and problem-solving, to 162 Grades 3 to 4 students with DLD and no significant difference in reading comprehension in post-test compared to the waiting control group.

Overall, there were limited findings regarding how extended discourse interventions affect reading comprehension. There is a need to investigate the potential difference in procedure (i.e., the order of technique delivery) given the wide scope of the intervention programs.

### 3.4. Reading comprehension outcome characteristics

Nineteen of the 24 included studies (79 %) adopted standardized reading comprehension tests to measure intervention outcomes. Four studies (17 %) used experimental reading comprehension tasks to measure reading outcomes in which all involved reading narrative text(s) and answering literal and inference questions verbally. None of the experimental reading comprehension tasks provided reliability information. One study adopted reading book level as the reading comprehension intervention measure.

### 3.5. Risk of bias assessment

Sixteen of the included studies (67 %) reported measures in ensuring treatment fidelity, including observation and feedback, interrater review, checklist review, etc. Three different risk-of-bias assessments were used to evaluate the included studies based on their intervention design (Supplementary Material 2 shows the full results). Quality Assessment of Controlled Intervention Studies was used for 14 studies, including RCT studies and studies with a control group. Most studies had a similar baseline across groups (13/14; 87%), low dropout rate (i.e., less than 20 %, 14/14; 100%), and valid and reliable outcome measures (11/14; 79 %). However, only half of the studies had indicated blinded assessment (7/14), and only two of them (14 %) reported power analysis to justify the effect size. Four of five RCT studies included (80 %) provided adequate information on randomization method and concealed treatment allocation.

Quality Assessment Tool for Before-After (Pre-Post) Studies With No Control Group was adopted for the three included group design studies. All of them had low dropout rates (i.e., less than 20 %), and statistical tests were used to report the pre- and post-treatment change. Two of them (66.67 %) had a clear definitions of inclusion criteria and valid and reliable outcome measures. However, none of them reported on blind assessment and power analysis to justify the effect size.

The Quality Assessment Tool for Case Series Studies was adopted for the seven included single-case studies. All of them had clear research questions. Over half of them had a clearly described intervention (5/7; 71%), valid and reliable outcome measures (5/7; 71 %), comparable subjects (4/7; 57 %), and well-described results (4/7; 57 %). However, only two of them (29 %) had adequate length of follow-up.

## 4. Discussion

This review of reading comprehension interventions for children with DLD included 24 studies spanning 24 years. This field of intervention research is dominated by group design and single-case studies with only one intervention that have been replicated (Wright et al., 2015). Nonetheless, the current review shows that 19 out of 24 studies reported some forms of significant improvement in reading comprehension, and all studies align with the AVR model. This pattern of findings is promising and, with further research, suggests that strong evidence-based practices for children with DLD can be developed. It should be noted that most of the studies were with young children from elementary school-age, and code-based interventions also had significant improvement in reading comprehension. Threats to validity were present, including variations in dosage and intensity of the interventions and measurement approaches, which would limit the clinical application of the findings, should be considered in future research. Despite these reservations, different reading comprehension interventions showed significant improvement, and future research should investigate the efficacy and effectiveness of these interventions on a larger scale.

### 4.1. Findings in the context of the AVR theory

We were able to classify all included studies into one or more components in the AVR based on the target skill(s) of the intervention (Duke & Cartwright, 2021). The intervention programs included one or more of the following components based on their treatment focus: self-regulation (with a focus on reading strategies instruction), word recognition (with a focus on phonological-level instruction), bridging process (with a focus on vocabulary knowledge and morphological awareness), and language comprehension (usually addressing extended discourse as the treatment target). Given the variations of participants and intervention features, it is difficult to draw strong conclusions evaluating the effectiveness of reading comprehension interventions. However, based on the available studies, all interventions targeting vocabulary knowledge and morphological awareness (categorized as the bridging process) had significant positive gains (Katz & Carlisle, 2009; McLeod & Apel, 2015; Rousseau et al., 1993; Staudt, 2009). This is consistent with findings from a meta-analysis (Burns et al., 2023) that interventions targeting bridging processes had the largest effect size compared to interventions targeting other components in typically developing children and children with reading difficulties. This is likely because the bridging process accounts for more shared variance of word recognition and listening comprehension than each of their variances (Foorman et al., 2020; Lonigan et al., 2018), leading to greater improvements in reading comprehension.

The relation between target skills in reading comprehension interventions and age remains unexplored. The included studies with adolescent participants focused on either strategy use or vocabulary knowledge, while the included studies with child participants focused on various aspects of reading comprehension skills. This is in line with the rope model of reading (Scarborough, 2001), which suggests that word recognition becomes more automatic and language comprehension skills become more strategic across ages for skilled reading comprehension. Thus, reading comprehension intervention in adolescents might focus on developing strategies for extended discourse skills, and that in children might focus on various aspects of reading skills. However, the included studies mostly focused on young children with DLD: they mostly focused on the *learning to read* stage, and less was understood on later stages of reading comprehension, including *reading to learn*, multiple viewpoints, and construction and reconstruction (Chall, 1983). The later stages are more complex and have a strong relation with academic success, and should not be ignored for the DLD population. More studies are needed to understand (1) the appropriate age for introducing reading comprehension strategies and (2) the extended discourse support on reading comprehension in adolescents with DLD. Children with DLD showed lower performance on executive functioning skills compared to age-matched peers (e.g., Blom & Boerma, 2020). Utilizing various reading comprehension strategies which can alleviate the lower executive function in children with DLD which in turn lower the linguistic processing demands. Thus, it is important to investigate the appropriate age for various reading comprehension strategies to prevent overloading their cognitive demands during reading comprehension. In addition, most of the extended discourse interventions focused on children in early school-age only, and the importance of extended discourse in reading comprehension increases over time when word recognition becomes automatic (Nation & Snowling, 2004). Given that DLD is a lifelong condition (Georgan et al., 2023), extended discourse support or intervention in adolescents might be effective for children with DLD to understand more complex texts.

It should also be noted that seven sub-components of the AVR had no studies included in the current review (motivation and engagement, alphabetic principle, reading fluency, graphophonological-semantic cognitive flexibility, cultural and other content knowledge, reading-specific background knowledge, and theory of mind). However, previous studies show that these components are related to reading comprehension skill in children with DLD (Lam et al., 2024), and interventions on these components can improve reading comprehension in both typically developing and struggling readers (Duke & Cartwright, 2021).

### 4.2. Threats to validity to reading comprehension intervention

One of the most important limitations that may hinder the ability to generalize the findings of the current scoping review is variations in dosage and intensity of the interventions. The included studies have a wide range of dosages and intensities, and no studies have examined the optimal amount of intervention needed for a significant effect size on reading comprehension performance. Thus, it remains unclear whether the non-significant results are due to non-optimal dosage and intensity.

Another area of limitation is the measurement approaches employed in the reviewed studies. Although the majority of studies used standardized reading comprehension tasks, such assessments may not be able to fully capture the complexities of the reading development of children with DLD. Children’s performance in reading comprehension varies by a number of factors, including individual factors (i.e., age, linguistic backgrounds, various skills involved in the reading comprehension process), text features (i.e., differential linguistic and structural features in various genres), and question types (e.g., literal and inferential comprehension questions; Kim & Petscher, 2021). The more specific, nuanced gradations that criterion-referenced measures can capture could be ignored when using general standardized reading comprehension measures (McCauley & Swisher, 1984). Standardized measures thus make it challenging to understand the multifaceted nature of reading comprehension development for this group of children, whose development and demands in various aspects of language and literacy skills likely vary. Therefore, it is important to use reading comprehension measures with ecological validity in addition to standardized assessments to examine the performance of various key component skills of reading, and how the performance differs by genres and question types. It is also worth noting that three of the studies utilized experimental tasks without reporting reliability information. Furthermore, interventions were often carried out without blinding of assessment, and only a small number of studies conducted follow-up assessments to evaluate the sustainability of intervention effects. This limitation restricts our understanding of the long-term impact of reading comprehension interventions in children with DLD.

### 4.3. Gaps in the literature

A gap in the literature is the lack of representation of multilingual children with DLD or children speaking another language as first language. Among the studies we reviewed, there was a strong focus (88 % of the studies) on monolingual English-speaking participants. This may have overlooked the diverse experiences and demands of multilingual children with DLD and children who speak a language other than English as their first language. The diverse linguistic backgrounds among multilingual children with DLD necessitate tailored and culturally sensitive reading comprehension interventions. Instruction that draws on children’s diverse linguistic backgrounds and funds of knowledge can facilitate their literacy development. Neglecting the needs of this population may risk leaving a large portion of the DLD population underserved. Future studies should examine the features of reading comprehension interventions that can cater to the needs of multilingual readers and readers with various first languages.

Furthermore, the optimal intervention dosage for reading comprehension interventions needs to be explored. The intervention dosage for various areas to achieve optimal treatment effectiveness is important for clinical practice. Future studies can consider examining the optimal intervention dosage, such as session length, dose frequency, total intervention duration, cumulative intervention intensity, and so on, based on the types of intervention (Zeng et al., 2012). This can help examine the outcome of interventions while balancing the economic or resource concerns. In addition, the frequency of the intervention (i.e., spacing effect and massed learning) might also be a topic for future research, given that reading comprehension is a skill that takes time to develop, and children with DLD may thus need continuous support in this area.

### 4.4. Clinical implications

Although the nature of a scoping review limits our ability to provide definitive instructional practice recommendations regarding the feasibility, appropriateness, and effectiveness of specific interventions (Munn et al., 2018), there are two major clinical implications. First, the current review highlights a wide range of treatment approaches for improving reading comprehension in children with DLD. Interventions targeting different components of the AVR framework, including oral language, decoding, bridging processes, and active self-regulation, showed significant and positive outcomes for individuals with DLD. This suggests that clinicians can consider a variety of approaches when designing interventions. Second, clinicians should take into account both the individual’s characteristics (e.g., language profile, developmental stage, and comorbidities) and dosage-related information (e.g., frequency, intensity, and duration) when tailoring interventions. Considering these factors may help maximize effectiveness of interventions for individuals with DLD.

## 5. Conclusion

The current study reviewed evidence regarding interventions targeting reading comprehension among individuals with DLD, with reference to the AVR framework. The 37 interventions that were reviewed aligned with many of the components of the AVR framework and reported mostly positive outcomes in reading comprehension, despite variations in design, dosage, and measures. By mapping intervention characteristics and identifying gaps in the current literature, this review lays the groundwork for both future empirical research and systematic reviews, which can more precisely evaluate intervention effectiveness and inform clinical practice.

## Supplementary Material

1

2

Supplementary material associated with this article can be found, in the online version, at doi:10.1016/j.jcomdis.2025.106608.

## Figures and Tables

**Table 1:** Summary of Interventions in the Active Self-regulation Component.

Study	Participants Characteristics			Intervention Characteristics						Intervention Outcomes	
	Language Ability Status	Mean Age	*N*	Name	Target skill(s)^a^	Duration	Dosage (hour)	Intensity (minute/week)	Frequency	Measures	Reading comprehension outcome
Ritter, Colson et al. (2013)	LI	9;4	49	Interactive metronome (IM) and language and reading intervention	Executive functions, decoding, vocabulary knowledge, phonological awareness	4 weeks	52	780	4 to 5 times a week	GORT reading comprehension subtest	Significant treatment effect across group and higher gain in the IM group
				Language and reading intervention	Decoding, vocabulary knowledge, phonological awareness	4 weeks	48	720	4 to 5 times a week		
Wright et al. (2015)	LI	Range: 12;2 – 14; 9	28	Multiple reading comprehension strategies (with replication)	Strategies use, Motivation and engagement, content knowledge	4 weeks	8	120	Twice a week	YARC	Significant difference pre/post-intervention with large effect size
Takala (2006)	SLI	fourth and six graders	50	Reciprocal strategy teaching	Strategies use	5 weeks	7.5–11.25	90–135	Weekly	Experimental reading tasks	Improvement but no statistical analysis
McCartney et al. (2015)	LI	7.58	18	Reading comprehension strategies intervention	Strategies use	A school year	NR	NR	NR	WIAT	Significant difference pre/post-intervention with large effect size
Lukin and Estraviz (2010)	Speech Language impairment	NR	6	Reading Recovery	Strategies use, print concept	18–27 weeks	NR	NR	4 times a week	Book level	Improvement but no statistical analysis
Sanabria et al. (2022)	Weak language skills	9.13	56	Enhanced Moved by Reading to Accelerate Comprehension in English (EMBRACE)	Strategies use, vocabulary knowledge	4 weeks	8	120	4 times a week	Gates-MacGinitie Reading Test (MacGinitie & MacGinitie, 2000)	Non-significant overall intervention effect
				Same intervention but no manipulation of the images	Vocabulary knowledge						
Joffe et al. (2007)	SLI	9.6	9	Imagery intervention	Strategies use	3 weeks	2.5	30–60	1 to 2 times a week	Experimental Reading Task	Significant improvement on literal questions but not for inferential questions
Green and Roth (2013)	language disroder	10;6	1	Inferential comprehension intervention	Strategies use, verbal reasoning	8 weeks	4	30–40	Weekly	Gates-MacGinitie Reading Test (MacGinitie & MacGinitie, 1989)	Improvement but no statistical analysis
Starling et al. (2012)	LI	13.25	43	Instructional language modification technique	Strategies use, vocabulary knowledge	10 weeks	8.33	50	Weekly	WIAT	Significant pre/post improvement in written expression and listening comprehension

*Note*.

a Identified with reference to Active View of Reading (Duke & Cartwright, 2021). For studies that have more than one intervention, the upper one indicates the target intervention. LI = language impairment, SLI = specific language impairment, NR = not reported, GROT = Gray Oral Reading Test–Fourth Edition (Wiederholt & Bryan, 2001) YARC = York Assessment of Reading for Comprehension (Snowling et al., 2010), WIAT = Wechsler Individual Achievement Test, Second UK Edition for Teachers Reading Comprehension Scale (Wechsler 2006).

**Table 2:** Summary of Interventions in the Word Recognition Component.

Study	Participants Characteristics			Intervention Characteristics						Intervention Outcomes	
	Language Ability Status	Mean Age	*N*	Name	Target skill(s)^a^	Duration	Dosage (hour)	Intensity (minute/week)	Frequency	Measures	Reading comprehension outcome
Crowe (2003)	Language learning disabilities	9.94	8	Traditional decoding-based feedback	Decoding skills, phonological awareness, phonetics knowledge, strategies use	5 weeks	12	100	Twice a week	GORT-R	Communicative reading strategies had higher reading comprehension outcome than the traditional decoding-based group
				Communicative reading strategies	Language structure, strategies use						
Crowe (2005)	Language learning disabilities	10.04	12	Traditional decoding-based feedback	Decoding skills, phonological awareness, phonetics knowledge, strategies use	6 weeks	12	120	Twice a week	GORT-R	Communicative reading strategies had higher reading comprehension outcome than the traditional decoding-based group
				Communicative reading strategies	Language structure, strategies use						
Loeb et al. (2009)	LI	7;7	103	Fast for word language intervention	Phonological awareness, phonetics knowledge, language structure	6 weeks	50	500	5 times a week	Woodcock Reading Mastery Tests—Revised (Woodcock, 1987)	No significant difference among groups with medium effect size of gain
				Computer-assisted language intervention	Phonological awareness, phonetics knowledge, language structure						
				individualized language intervention	Phonological awareness, phonetics knowledge, language structure						
Pokorni et al. (2004)	Language and reading deficits	8.67	54	Fast ForWord	Phonological awareness, language structure	4 weeks	60	900	5 times a week	WLPB-R	No significant difference among groups
				Earboics Step2 intervention	Phonological awareness						
				LiPS	Phonological awareness						
Ritter, Park et al. (2013)	LI	8.04	64	Phonological Awareness Intervention	Phonological awareness	12 weeks	6	30	Twice a week	WDRB-R	Significant improvement on reading comprehension compared to control condition
Bowyer-Crane et al. (2008)	Low language skills	4;9	152	Phonology with Reading	Phonological awareness, alphabetic principle, phonic knowledge, reading fluency	20 weeks	83.3	250	5 times a week	NARA and GORT	No significant difference on reading comprehension outcome
				Oral language program	Vocabulary knowledge, listening comprehension, verbal reasoning						
Kouri (2016)	LI	Range = 7;5–9;11	15	Guided reading (graphophonemic based feedback)	Phonological awareness, phonics knowledge, decoding skills	28 weeks	NR	NR	Weekly	experimental reading task	No significant difference across three conditions
				Guided reading (meaning based feedback)	language structure						
				Guided reading (mixed feedback)	Phonological awareness, phonics knowledge, decoding skills, language structure						
Buck et al. (1988)	LI	Range = 7;1 – 10;5	8	Home-based tutoring program	Recognition of word at sight, print knowledge, phonic knowledge, writing	NR	15.5	60–90	Weekly	Woodcock Reading Mastery Test (Woodcock, 1973)	Significant improvement in reading comprehension

*Note*.

a Identified with reference to Active View of Reading (Duke & Cartwright, 2021). For studies that have more than one intervention, the upper one indicates the target intervention. LI = language impairment, NR = not reported, GROT = Gray Oral Reading Test–Fourth Edition (Wiederholt & Bryan, 2001), NARA = Neale Analysis of Reading Ability II (Neale, 1997), WLPB-*R* = Four subtests of the Woodcock Language Proficiency Battery–Revised (Woodcock, 1991), WDRB-*R* = Woodcock Diagnostic Reading Battery–Revised (Woodcock, 1997), WIAT = Wechsler Individual Achievement Test, Second UK Edition for Teachers Reading Comprehension Scale (Wechsler 2006).

**Table 3:** Summary of Interventions in the Vocabulary Knowledge Component.

Study	Participants Characteristics			Intervention Characteristics						Intervention Outcomes	
	Language Ability Status	Mean Age	*N*	Name	Target skill(s)^a^	Duration	Dosage (hour)	Intensity (minute/week)	Frequency	Measures	Reading comprehension outcome
Rousseau et al. (1993)	Speech and language deficits	12.0	5	Key words and/or listening preview	Vocabulary knowledge	10 weeks	NR	NR	5 times a week	Experimental reading tasks	Showed improvement but no statistical analysis
Staudt (2009)	Language disorder	fourth grade	1	Teaching vocabulary knowledge	Vocabulary knowledge, phonological awareness, alphabetic principle	One school year	NR	NR	Every school day	Gates-MacGinitie Reading Test (MacGinitie & MacGinitie, 2000)	Showed improvement
Katz and Carlisle (2009)	Language difficulties	Range = 9;4–9;11	3	Close Reading Program	Morphological awareness, strategies use	12 weeks	12	60	Twice a week	Woodcock-Johnson Psychoeducational Battery—Revised (Woodcock & Johnson, 1989)	Showed improvement with medium to large effect sizes
McLeod & Apel, 2015	LI	6;7	1	Morphosyntax awareness intervention	Morphological awareness	7 weeks	14.5	105–140	3 to 4 times a week	Test of Silent Reading Efficiency and Comprehension (Wagne et al., 2010)	Showed improvement but with percentage overlap

*Note*.

a Identified with reference to Active View of Reading (Duke & Cartwright, 2021). For studies that have more than one intervention, the upper one indicates the target intervention. LI = language impairment, NR = not reported.

**Table 4:** Summary of Interventions in the Extended Discourse Component.

Study	Participants Characteristics			Intervention Characteristics						Intervention Outcomes	
	Language Ability Status	Mean Age	*N*	Name	Target skill(s)^a^	Duration	Dosage (hour)	Intensity (minute/week)	Frequency	Measures	Reading comprehension outcome
Fricke et al. (2013)	Weak oral language skills	4;0	180	Nuffield Early Language Intervention	Expressive language, verbal reasoning, listening comprehension, vocabulary knowledge, phonological awareness	30 weeks	42.5	45–120	3 to 5 times a week	YARC	Significant higher performance compared to waiting control
Fricke et al. (2017)	Weak oral language skills	3.84	394	Nuffield Early Language Intervention	Expressive language, verbal reasoning, listening comprehension, vocabulary knowledge, phonological awareness	30 weeks	47.75	45–120	3 times a week	YARC	No significant difference between two conditions and waiting control
						20 weeks	37.75	45–120	3 times a week		
Revees et al. (2019)	Low-average communication skills	8;2	162	Talk Boost KS2	Language structure, vocabulary knowledge, strategy use, expressive language	8 weeks	16	120	3 times a week	YARC	No significant difference compared to waiting control

*Note*.

a Identified with reference to Active View of Reading (Duke & Cartwright, 2021). For studies that have more than one intervention, the upper one indicates the target intervention. YARC = York Assessment of Reading for Comprehension (Snowling et al., 2010).

## Data Availability

The complete dataset is available in the replication materials (https://osf.io/5vs7n).
