# Supplementary material for: Reading comprehension intervention in populations with developmental language disorder: A scoping review
Source: J Commun Disord. Author manuscript; Available in PMC 2026 Jun 23. (PMC13286734; doi:10.1016/j.jcomdis.2025.106608)
Supplement: 1 [file NIHMS2187284-supplement-1.docx]

Supplementary Material 1.

Compete Search String.

(“Reading comprehension”) AND (“intervention” OR “teaching methods” OR “therapy” OR “instruction” OR “treatment” OR “program”) AND (“Communication problems” OR “Communication needs” OR “Language problems” OR “Language difficulties” OR “Communication difficulties” OR “Language needs” OR “Specific language impairment” OR “Communication delay” OR “Language impairment” OR “Language disorder” OR “Language delay” OR “Communication disorder” OR “Communication impairment” OR “Language disability” OR “Developmental language disorder” OR “Speech and language difficulties” OR “Speech and language disorder” OR “Speech and language problems” OR “Communication disability” OR “Developmental aphasia” OR “Speech and language impairment” OR “Speech and language delay” OR “Developmental dysphasia” OR “Language learning needs” OR “Speech/language impairment” OR “Language learning difficulties” OR “Language learning problems” OR “Developmental language delay” OR “Developmental language impairment” OR “Language learning disability” OR “Speech/language disorder” OR “Speech, language and communication needs” OR “Speech/language problems”)
