# Supplementary material for: Reading comprehension intervention in populations with developmental language disorder: A scoping review
Source: J Commun Disord. Author manuscript; Available in PMC 2026 Jun 23. (PMC13286734; doi:10.1016/j.jcomdis.2025.106608)
Supplement: 2 [file NIHMS2187284-supplement-2.docx]

Supplementary Material 2.

Summary Statistics of Risk of Bias Assessment.

Quality Assessment of Controlled Intervention Studies (*n* = 14)

|  | Yes | No | Not reported | Can’t Determine and Not Applicable |
| --- | --- | --- | --- | --- |
| 1. Was the study described as randomized, a randomized trial, a randomized clinical trial, or an RCT? | 5 | 9 | 0 | 0 |
| 2. Was the method of randomization adequate (i.e., use of randomly generated assignment)? | 4 | 9 | 1 | 0 |
| 3. Was the treatment allocation concealed (so that assignments could not be predicted)? | 5 | 8 | 1 | 0 |
| 4. Were study participants and providers blinded to treatment group assignment? | 0 | 10 | 4 | 0 |
| 5. Were the people assessing the outcomes blinded to the participants' group assignments? | 7 | 3 | 4 | 0 |
| 6. Were the groups similar at baseline on important characteristics that could affect outcomes (e.g., demographics, risk factors, co-morbid conditions)? | 13 | 1 | 0 | 0 |
| 7. Was the overall drop-out rate from the study at endpoint 20% or lower of the number allocated to treatment? | 14 | 0 | 0 | 0 |
| 8. Was the differential drop-out rate (between treatment groups) at endpoint 15 percentage points or lower? | 14 | 0 | 0 | 0 |
| 9. Was there high adherence to the intervention protocols for each treatment group? | 14 | 0 | 0 | 0 |
| 10. Were other interventions avoided or similar in the groups (e.g., similar background treatments)? | 8 | 0 | 6 | 0 |
| 11. Were outcomes assessed using valid and reliable measures, implemented consistently across all study participants? | 11 | 1 | 0 | 2 |
| 12. Did the authors report that the sample size was sufficiently large to be able to detect a difference in the main outcome between groups with at least 80% power? | 2 | 0 | 12 | 0 |
| 13. Were outcomes reported or subgroups analyzed prespecified (i.e., identified before analyses were conducted)? | 12 | 0 | 0 | 2 |
| 14. Were all randomized participants analyzed in the group to which they were originally assigned, i.e., did they use an intention-to-treat analysis? | 12 | 0 | 0 | 2 |

Quality Assessment Tool for Before-After (Pre-Post) Studies With No Control Group (*n* = 3)

|  | Yes | No | Not reported | Can’t Determine and Not Applicable |
| --- | --- | --- | --- | --- |
| 1. Was the study question or objective clearly stated? | 3 | 0 | 0 | 0 |
| 2. Were eligibility/selection criteria for the study population prespecified and clearly described? | 2 | 1 | 0 | 0 |
| 3. Were the participants in the study representative of those who would be eligible for the test/service/intervention in the general or clinical population of interest? | 2 | 0 | 0 | 1 |
| 4. Were all eligible participants that met the prespecified entry criteria enrolled? | 2 | 0 | 0 | 1 |
| 5. Was the sample size sufficiently large to provide confidence in the findings? | 0 | 0 | 3 | 0 |
| 6. Was the test/service/intervention clearly described and delivered consistently across the study population? | 2 | 0 | 0 | 1 |
| 7. Were the outcome measures prespecified, clearly defined, valid, reliable, and assessed consistently across all study participants? | 3 | 0 | 0 | 0 |
| 8. Were the people assessing the outcomes blinded to the participants' exposures/interventions? | 0 | 0 | 3 | 0 |
| 9. Was the loss to follow-up after baseline 20% or less? Were those lost to follow-up accounted for in the analysis? | 3 | 0 | 0 | 0 |
| 10. Did the statistical methods examine changes in outcome measures from before to after the intervention? Were statistical tests done that provided *p* values for the pre-to-post changes? | 3 | 0 | 0 | 0 |
| 11. Were outcome measures of interest taken multiple times before the intervention and multiple times after the intervention (i.e., did they use an interrupted time-series design)? | 1 | 2 | 0 | 0 |
| 12. If the intervention was conducted at a group level (e.g., a whole hospital, a community, etc.) did the statistical analysis take into account the use of individual-level data to determine effects at the group level? | 1 | 1 | 0 | 1 |

Quality Assessment Tool for Case Series Studies (*n =* 7)

|  | Yes | No | Not reported | Can’t Determine and Not Applicable |
| --- | --- | --- | --- | --- |
| 1. Was the study question or objective clearly stated? | 7 | 0 | 0 | 0 |
| 2. Was the study population clearly and fully described, including a case definition? | 5 | 2 | 0 | 0 |
| 3. Were the cases consecutive? | 4 | 0 | 0 | 3 |
| 4. Were the subjects comparable? | 4 | 0 | 0 | 3 |
| 5. Was the intervention clearly described? | 5 | 0 | 0 | 2 |
| 6. Were the outcome measures clearly defined, valid, reliable, and implemented consistently across all study participants? | 5 | 0 | 0 | 2 |
| 7. Was the length of follow-up adequate? | 2 | 5 | 0 | 0 |
| 8. Were the statistical methods well-described? | 3 | 0 | 0 | 4 |
| 9. Were the results well-described? | 4 | 1 | 0 | 2 |
